# Do policies that allow access to unregistered antimicrobials address the unmet need? Australia as a case study of a high-income country with universal healthcare

**DOI:** 10.1093/jacamr/dlae216

**Published:** 2025-02-27

**Authors:** Nadine T Hillock, Allen Cheng, Andrew Bowskill

**Affiliations:** School of Public Health, University of Adelaide, Adelaide 5005, Australia; Centre to Impact Antimicrobial Resistance, Monash University, Clayton 3800, Australia; Monash Infectious Diseases, Monash Health and School of Clinical Sciences, Monash University, Clayton 3168, Australia; Australian Antimicrobial Resistance Network (AAMRNet), MTPConnect, Brighton 3186, Australia

## Abstract

**Background:**

Ensuring timely and equitable access to effective and optimal antimicrobials is crucial for optimal patient care, to minimize the use of less appropriate treatment options and reduce the risk of antimicrobial resistance (AMR).

**Objectives:**

To determine the average time for new antibacterials to gain registration for use in Australia after obtaining marketing approval internationally, and to quantify the use of ‘new’ and older unregistered antimicrobials in Australian clinical practice between 2018 and 2023.

**Methods:**

Two data sources were utilized to estimate the usage of antimicrobials not registered for use in Australia. Annual hospital inpatient usage data were sourced from the National Antimicrobial Utilisation Surveillance Program (NAUSP) and data on Special Access Scheme (SAS) applications for unregistered antimicrobial was sourced from the Australian Government Department of Health and Aged Care.

**Results:**

Between 2018 and 2023 there were 36 131 applications to access unapproved antimicrobials in Australia. In 26.6% of cases, access to an unapproved antimicrobial was for the treatment of a critically ill patient. Levofloxacin, pyrazinamide, tetracycline and pristinamycin were the most frequently accessed unregistered antimicrobials. Applications for ‘new’ antibacterials increased from 55 in 2018 to 249 in 2023. Inpatient use of nine new antibacterials was reported in Australian hospitals in 2023, two registered and seven unregistered.

**Conclusions:**

Unapproved antimicrobials are frequently accessed by clinicians for patients unable to be treated with registered antimicrobials in Australia. Policy reform and economic incentives are required to support the registration of antimicrobials needed for otherwise untreatable infections and to ensure the sustainability of supply.

## Introduction

Ensuring timely and equitable access to effective and most appropriate antimicrobial treatment is crucial for optimal patient care and to minimize the risk of antimicrobial resistance (AMR).^1^ Increasingly, clinicians are encountering scenarios where patients have infections where few therapeutic options are available due to multidrug resistance. Additionally, a fundamental challenge jeopardizing efforts to manage AMR is lack of access to first-line antibacterials, either due to regulatory or economic barriers that make it commercially unviable for manufacturers, or shortages.^2^

Inappropriate antibacterial use is one of the key factors driving the development and spread of AMR. Ensuring that the optimal antibacterial is affordable and accessible for patients in a timely manner is an important strategy to minimize the use of inappropriate alternative drugs. Barriers to the availability and registration of new antibacterials that are needed for the treatment of otherwise untreatable, MDR infections have been highlighted previously.^3,4^ The WHO has highlighted the critical need for new antibacterial drugs to treat the increasing number of MDR infections globally.^5^ Although recent reviews show a small increase in the number of antibacterials in the clinical pipeline,^6–9^ the WHO acknowledges that these are insufficient to effectively meet the increasing need due to the ongoing emergence and spread of AMR.^5^ When new antibacterials are marketed, typically they are only accessible in larger, high-income countries such as the USA; however, previous studies have highlighted limited or delayed access in many other high-income countries such as Canada, Japan and many European countries.^10^ Global leaders have emphasized the urgent need to address the regulatory and economic barriers impeding the translation of new agents into clinical practice, and several high-income countries are exploring different incentive models to alleviate the economic risk for manufacturers.^11–13^ Antibacterial drug development is economically riskier for manufacturers than other medicines. The risk of resistance developing to a new potential antibacterial can impact efficacy in clinical trials, and therefore the potential sales. Treatment courses with antibacterials are usually short, resulting in lower economic returns than returns on medicines for chronic diseases. In addition, new antibacterials are generally restricted due to stewardship initiatives aimed at conserving the effectiveness by reducing use. In Australia, registration of a new drug is associated with substantial initial registration fees [initial fee of AU$284 669 (AU$56 844 application fee plus AU$227 825 evaluation fee); AU$284 669 = US$181 500 as at 12 December 2024], in addition to ongoing annual fees to maintain marketing approval.^14^ These fees apply to all new prescription medicines, including newly developed antimicrobials, as well as older off-patent medicines that are not currently approved in Australia. These fees alone may outweigh the economic returns possible for low-volume antimicrobials. For lower-income countries, the inability to afford new antimicrobials is a possible reason for delayed access.^15^

The legislation governing medicines access in Australia has allowance for the importation of unregistered medicines, known as the Special Access Scheme (SAS);^16^ however, this access pathway is associated with an increased liability and administrative burden on the prescriber, as well as an increased risk that supply of the medicine may be delayed or not available. Australia has a universal healthcare system (Medicare); however, unregistered medicines are not publicly funded in the community [on the Pharmaceutical Benefits Schedule (PBS)], nor are they usually funded by private insurers, increasing the risk that a patient may not be able to access the treatment outside the public hospital setting. Sponsors are obligated to ensure the availability of medicines registered with the Therapeutic Goods Administration (TGA) in Australia, and must notify the regulator if supply will be interrupted.^17^ This is not the case for unregistered medicines; there is no obligation to supply and therefore no assurance that they will be available in a timely manner when required, or if in fact they will be available at all.

Here we compared the registration status of new antimicrobials in Australia, in comparison with the USA and other international markets, and the utilization of these antimicrobials via access pathways for unregistered medicines. In addition, we aimed to quantify the use of all unregistered antibacterials, antifungals and antiparasitics in Australia between 2018 and 2023.

## Methods

The Australian Register of Therapeutic Goods (ARTG) was searched to determine registration dates for new antibacterials in Australia. For the purposes of this analysis, ‘new antibacterial’ refers to any newly developed antibacterial approved by the US FDA, the EMA or the Japanese Pharmaceuticals and Medical Devices Agency (PMDA) between 2013 and 2023.

Two datasets were used to estimate the usage of unregistered antimicrobials in Australia: (i) applications to access unregistered antimicrobials (excluding antivirals) via Australia’s SAS between January 2018 and December 2023 were retrieved from the Australian Government Department of Health and Aged Care via a Freedom of Information request: https://www.health.gov.au/resources/foi-disclosure-log. Data fields included the antimicrobial name, the date of application, the category of the application (Table 1), the product presentation, the route of administration, the state or territory, and the decision on the application (for Category B applications); and (ii) usage rates for adult inpatient use of new antimicrobials used in Australian hospitals, for January 2017 to December 2023, were retrieved via request from the National Antimicrobial Utilisation Surveillance Program (NAUSP).^18^

**Table 1.** Category definitions for the Australian SAS for unapproved medicines

Category	Definition
A	Patient is seriously ill with a condition from which death is reasonably likely to occur within a matter of months, or from which premature death is reasonably likely to occur in the absence of early treatment. Supply is not conditional on approval.
B	The patient does not meet Category A or C definitions, and clinical justification is required to support the application to use an unapproved product, including other treatment trialled/considered and the reason why an approved product is not appropriate. Approval of the request is required prior to supply.
C	A streamlined approval process for unapproved medicines included in a specified list^a^ of therapeutic goods with ‘an established history of use’ for specified indications.

^a^Inclusion of antimicrobials in the Category C list is managed by the TGA.

## Results

### Requests for unregistered antimicrobials via Australia’s SAS

**Figure 1.**
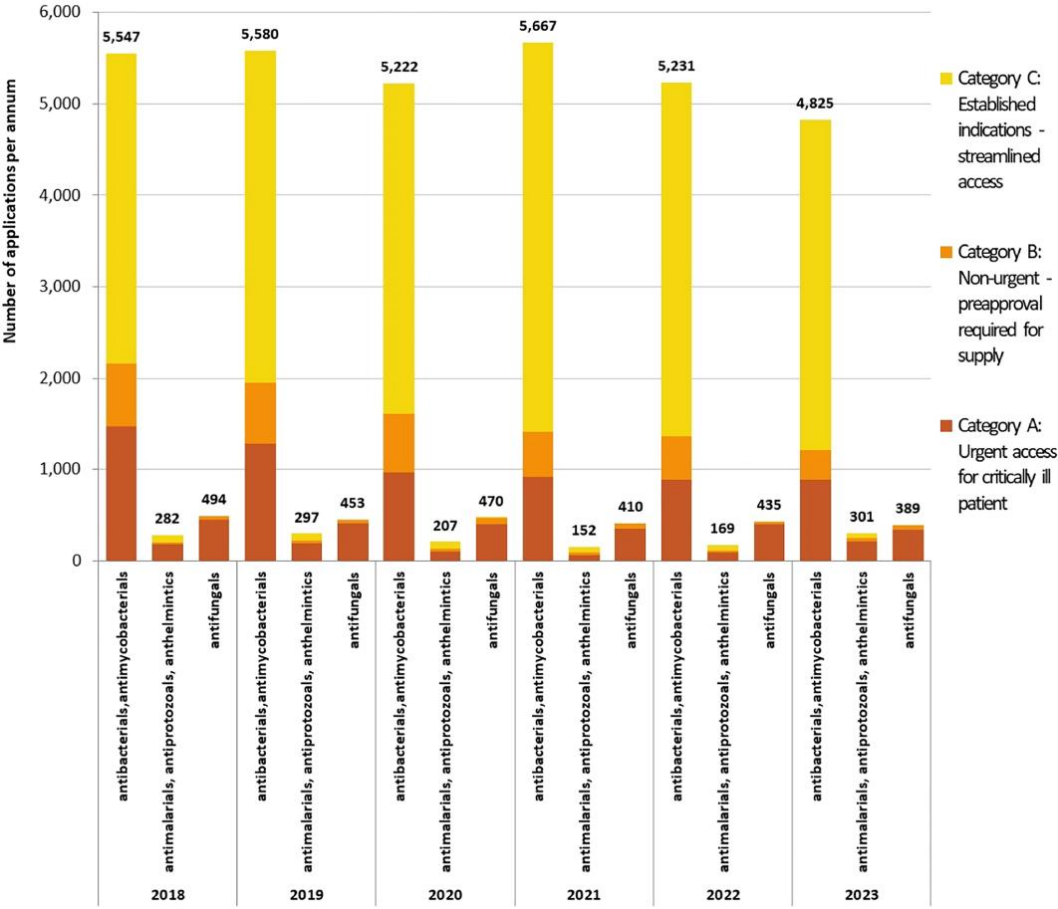
Annual applications for all antimicrobials via Australia’s SAS, by category of application and antimicrobial type, 2018–23. Category A = patient seriously ill (death likely within months), approval from TGA not required prior to supply (notification only); Category B = non-urgent, clinical justification required, approval from TGA required prior to supply; Category C = unregistered antimicrobials deemed to have ‘an established history of use’, approval from TGA not required (notification only). One hundred and seventy-three applications were withdrawn (13 Category A, 143 Category B and 17 Category C).

There were a total of 36 131 applications to access unregistered antimicrobials (excluding antivirals) between 2018 to 2023, of which 88.8% were antibacterials and/or antimycobacterials. Figure 1 illustrates the number of applications annually by category, and type of antimicrobial. Nine thousand, six hundred and ten applications (26.6%) were classified as Category A, where the unregistered antimicrobial was required for a critically ill patient. Three thousand, seven hundred and seventy-one (10.4%) were Category B applications and 22 750 (63.0%) were Category C. Of the 2742 Category B applications that require approval from the regulator prior to supply, 97% were approved within 20 days (median = 1 day, IQR: 1–4 days).

### Registration of new unregistered antibacterial agents with the TGA

New antibacterials (marketed internationally between 2013 and 2023) are listed in Table 2, along with the date of registration in Australia. Of the 23 unique antibacterial agents that have gained market authorization internationally since 2013, only two are listed on the ARTG in Australia: ceftazidime/avibactam and ceftolozane/tazobactam. Ceftolozane/tazobactam was registered for use in Australia 11 months after FDA approval in the USA. TGA registration for ceftazidime/avibactam occurred over 4 years (49 months) after FDA approval (Table 2).

**Table 2.** New antibacterials and their registration status in Australia

Antibacterial	WHO class	WHO code	Dosage form	Strength(s)	FDA registered	Date FDA registered	TGA registered	Date TGA registered
Aztreonam/avibactam	Monobactams	^a^	IV	1.5 g/0.5 g	No	—	No	—
Bedaquiline	Antimycobacterials	J04AK05	oral	20 mg, 100 mg	Yes	28/08/2013	No	—
Cefiderocol	Other cephalosporins and penems	J01DI04	IV	1 g/0.5 g	Yes	14/11/2019	No	—
Ceftazidime/avibactam	Third-generation cephalosporins	J01DD52	IV	2 g/0/5 g	Yes	25/02/2015	Yes	22/02/2019
Ceftobiprole	Other cephalosporins and penems	J01DI01	IV	500 mg	Yes	3/04/2024	No	—
Ceftolozane/tazobactam	Other cephalosporins and penems	J01DI54	IV	1 g/0.5 g	Yes	19/12/2014	Yes	4/11/2015
Contezolid	Oxazolidinone	^a^	oral	400 mg	No	—	No	—
Dalbavancin	Glycopeptides	J01XA04	IV	500 mg	Yes	23/05/2014	No	—
Delafloxacin	Fluoroquinolone	J01MA23	oral	450 mg	Yes	19/06/2017	No	—
Delafloxacin	Fluoroquinolone	J01MA23	IV	300 mg	Yes	19/06/2017	No	—
Delamanid	Antimycobacterials	J04AK06	oral	50 mg	No	—	No	—
Eravacycline	Tetracyclines	J01AA13	IV	50 mg, 100 mg	Yes	27/08/2018	No	—
Imipenem/cilastatin/relebactam	Carbapenems	J01DH56	IV	500 mg/500 mg/250 mg	Yes	16/07/2019	No	—
Lascufloxacin	Fluoroquinolone	J01MA25	oral	75 mg	No	—	No	—
Lefamulin	Other antibacterials	J01XX12	oral	600 mg	Yes	19/08/2019	No	—
Lefamulin	Other antibacterials	J01XX12	IV	150 mg	Yes	19/08/2019	No	—
Meropenem/vaborbactam	Carbapenems	J01DH52	IV	2 g/1 g	Yes	29/08/2017	No	—
Omadacycline	Tetracyclines	J01AA15	oral	150 mg	Yes	2/10/2018	No	—
Omadacycline	Tetracyclines	J01AA15	IV	100 mg	Yes	2/10/2018	No	—
Oritavancin	Glycopeptides	J01XA05	IV	400 mg, 1.2 g^b^	Yes	6/08/2014	No	—
Plazomicin	Aminoglycosides	J01GB14	IV	500 mg	Yes	25/06/2018	No	—
Pretomanid	Antimycobacterials	J04AK08	oral	200 mg	Yes	21/12/2022	No	—
Sarecycline	Tetracyclines	J01AA14	oral	60 mg, 100 mg, 150 mg	Yes	1/10/2018	No	—
Sulbactam/durlobactam	β-Lactamase inhibitors	^a^	IV	1 g/1 g	Yes	23/05/2023	No	—
Telavancin	Glycopeptides	J01XA03	IV	250 mg, 750 mg	Yes	21/06/2013	No	—
Tedizolid phosphate	Other antibacterials	J01XX11	oral	200 mg	Yes	20/06/2014	No	—
Tedizolid phosphate	Other antibacterials	J01XX11	IV	200 mg	Yes	20/06/2014	No	—

Fidaxomicin was registered in Australia in 2013; however, it fell outside the definition of ‘new’ antimicrobial for this analysis as it was approved by the TGA in 2011.

^a^Not assigned by the WHO.

^b^1.2 g strength listed by FDA in 2021; aztreonam/avibactam and delamanid have market authorization in Europe, contezolid in China, and lascufloxacin in Japan.

### Requests to access new unregistered antimicrobials

**Figure 2.**
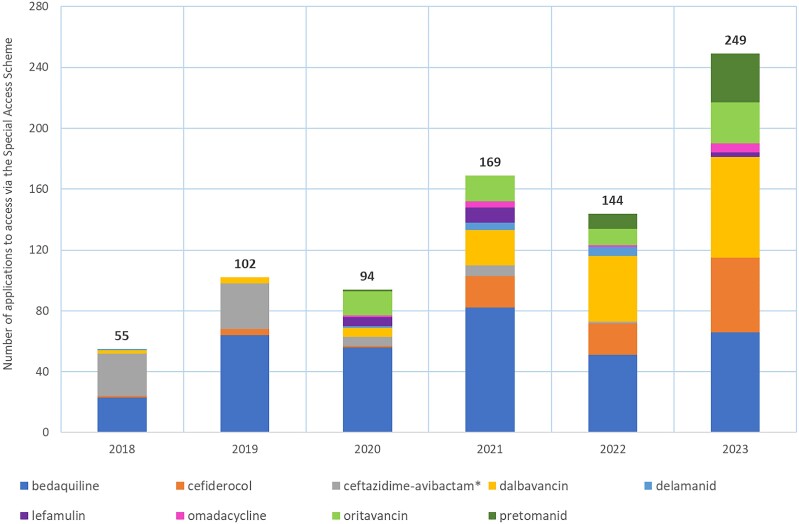
Annual applications to access newly developed antibacterials via the SAS, 2018–23. *Ceftazidime/avibactam was listed on the ARTG in February 2019.

The number of applications to access new, unregistered antimicrobials per annum are shown in Figure 2. Bedaquiline, dalbavancin and cefiderocol were the most frequently requested new antimicrobials; in 2023 there were 66 applications to access both bedaquiline and dalbavancin, and 49 requests for cefiderocol. The annual number SAS applications for new antibacterials for the period 2018–23 by Australian state or territory are provided in Figure S1 (available as Supplementary data at *JAC-AMR* Online).

### Requests to access older unregistered antimicrobials

**Figure 3.**
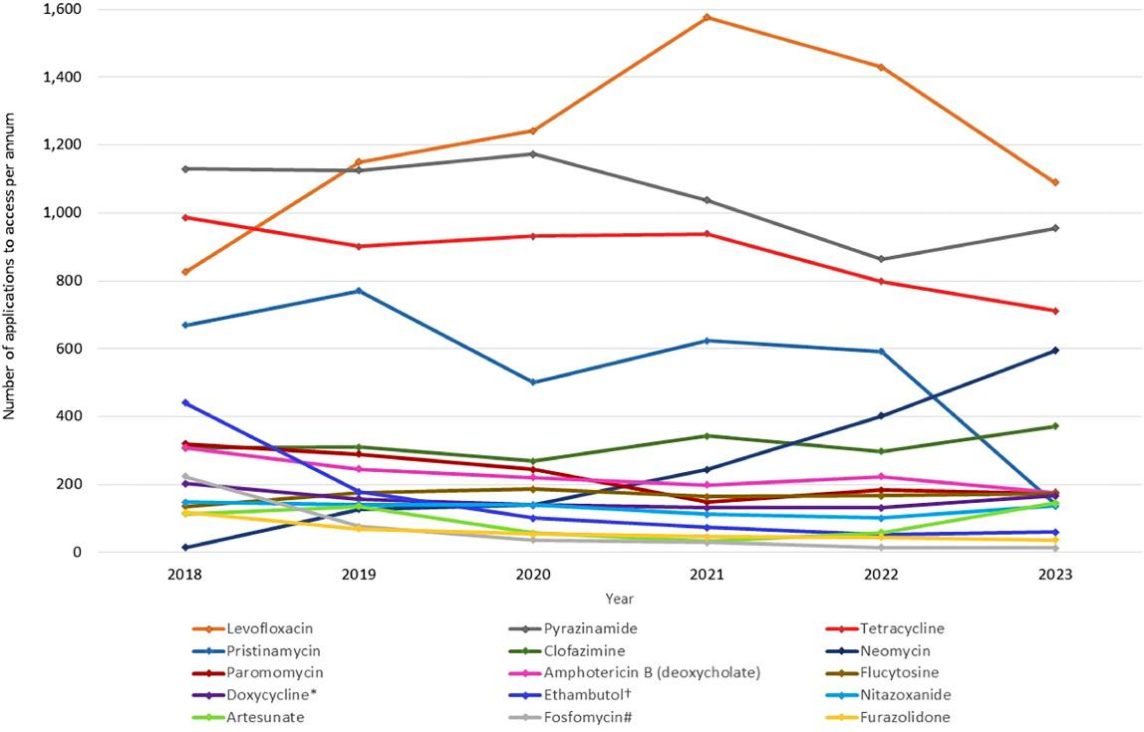
Most frequently accessed unregistered antimicrobials (annual SAS applications, 2018–23). *Doxycycline injection for intralesional use; ^†^ethambutol injection and 100 mg tablets; #fosfomycin injection, capsules/tablets.

Levofloxacin, which is included in Australian guidelines a part of salvage therapy for *Helicobacter pylori* infections, was the most frequently accessed unapproved antimicrobial in Australia between 2018 and 2023. This was followed by pyrazinamide, a first-line recommended treatment for TB, and tetracycline, also recommended as part of quadruple therapy for the salvage therapy of *H. pylori* infections not responsive to first-line treatment. Pristinamycin was the fourth most commonly used unregistered antibacterial. On average, there were 631 applications per year for pristinamycin from 2018 to 2022; however, in 2023 there was a global shortage of this antibacterial, which is reflected in the dramatic drop in use in 2023. None of the new antibacterials marketed internationally since 2013 were included in the 15 most frequently accessed unregistered antibacterials in Australia (Figure 3).

Of the most frequently accessed unregistered antimicrobials, oral neomycin use has increased the most significantly, with just 15 applications in 2018 and 595 applications in 2023.

Tinidazole was removed from the ARTG in 2019 after the last remaining sponsor withdrew the drug from the Australian market due to it not being economically viable to continue supply. Since then, there have been an average of 41 applications per year for the 4 years 2020 to 2023.

### Urgent SAS Category A requests

Category A applications are, by definition, used for patients who are critically ill, where death is imminent within months. Conventional amphotericin B was the antimicrobial most frequently requested via category A between 2018 and 2023, with multiple routes utilized including inhalation, ophthalmic, intravesical and IV administration. Antimicrobials accessed via Category A are often used to treat serious infections such as TB, malaria or MDR systemic infections, which can be fatal if access to effective treatment is not possible. Antimicrobials accessed via Category A are provided in Table S1.

### Use of unregistered antimicrobials in Australian hospitals

**Figure 4.**
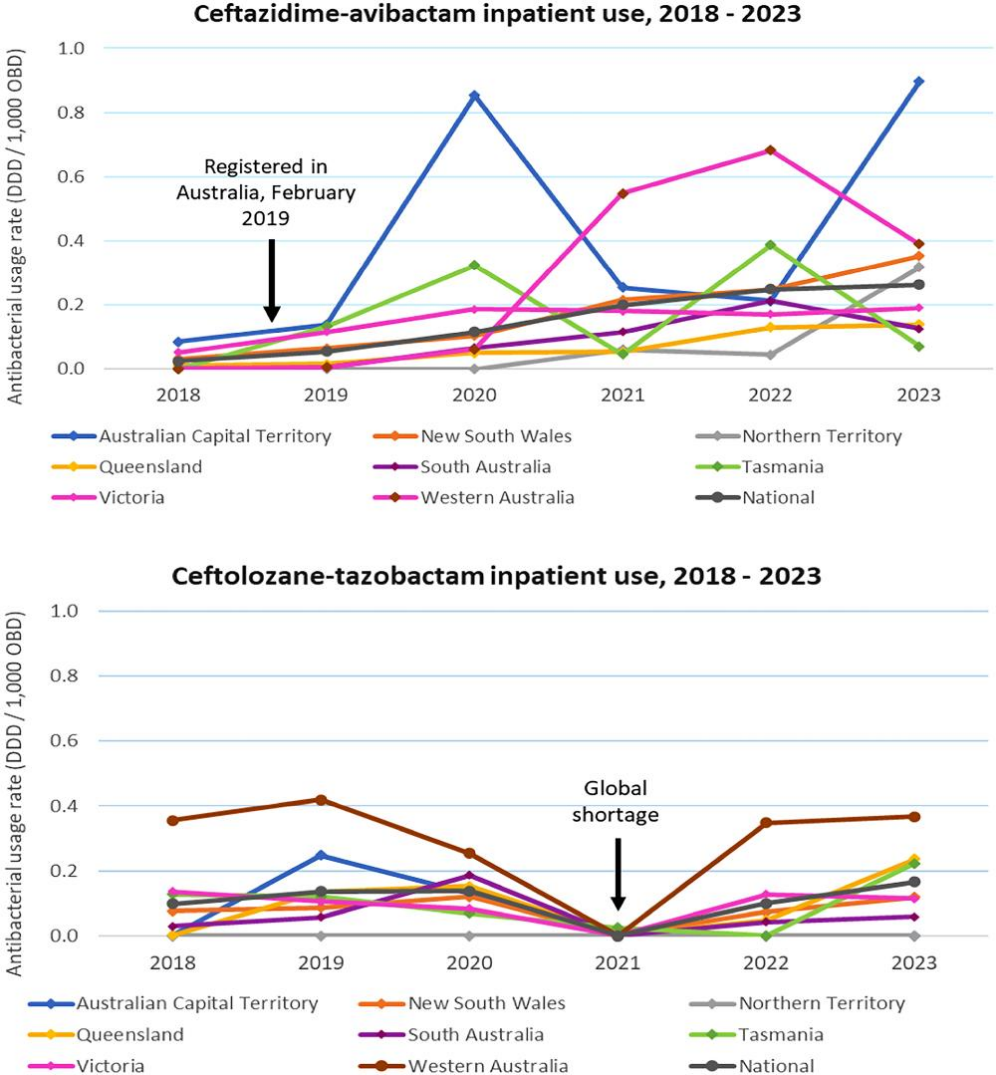
Hospital inpatient use (adults only) of newly developed antibacterials registered with the TGA* by Australian state or territory, NAUSP-contributor hospitals, 2018–23. *Registered in Australia between 2013 and 2023; OBD, occupied bed days.

Newly developed antibacterials entering clinical practice are predominantly used in the hospital setting to treat infections with MDR organisms. To estimate the utilization of new antibacterials in Australian hospitals, inpatient usage data for new antibacterials were retrieved from the NAUSP database on 22 July 2024. Nine new antibacterials were utilized by NAUSP-contributing hospitals in 2023, including seven not registered on the ARTG (Table S2). Annual inpatient usage rates (DDD/1000 occupied bed days) for the two new antibacterials registered on the ARTG are shown in Figure 4 for each of the Australian states and territories. There was a global shortage of ceftolozane/tazobactam in 2021 and no usage was reported. Ceftazidime/avibactam was registered in Australia in February 2019; usage in 2018 was negligible.

## Ethics

This research was considered to be negligible-risk research and exempt from HREC review as it satisfied both the conditions of: ‘It is negligible risk research: there is no foreseeable risk of harm or discomfort and any foreseeable risk is no more than inconvenience’ and ‘It involves the use of existing collections of data or records that contain only non-identifiable data about human beings’.

## Discussion

It is not uncommon in Australia for prescribers to require access to an antimicrobial that is not currently listed on the ARTG, the public database of therapeutic goods approved for supply. Although the annual requests to access unregistered antimicrobials fell over the 6 year period (from 6323 in 2018 to 5515 in 2023), it is noteworthy that this equates, on average, to 15–16 patients per day requiring access to an antibacterial that is not registered for use in Australia.

The majority of requests were to access older, off-patent antimicrobials that are available internationally but not approved for use in Australia. However, applications to access newly developed antibacterials to treat MDR infections are increasing (Figure 2). Despite the increase, the numbers of patients are still low (<50 patients per drug per year) and insufficient for pharmaceutical manufacturers to justify the expenses associated with registering and marketing these new drugs in Australia. Manufacturers are not obliged to have supplies of unregistered medicines available and therefore the ability to access these antibacterials in a timely and equitable manner is not assured. Additionally, the newer unregistered antimicrobials are used to treat life-threatening conditions such as sepsis, therefore a delay in procuring treatment can have a significant impact on survival. Registered medicines must meet strict quality standards enforced by the TGA; however, for unapproved medicines that are imported via the SAS pathway there is no guarantee of quality.

Orphan drug designation entitles a waiver of application and evaluation fees imposed by the TGA; however, antibacterials in general do not meet the definition if there are other antimicrobials registered that could potentially treat a particular infection, even if those registered antibacterials are not optimal from a stewardship perspective. Orphan status is designated by disease or clinical indication, not on the basis of susceptibility of a pathogen to the antibacterial.^19^ With some modification, the orphan drug access pathway could be explored for novel antimicrobials. Many older antimicrobials are very cheap and arguably undervalued in comparison with other medicines, especially considering their public health impact. The complexities associated with placing a price on antimicrobials have been highlighted previously.^4^

The recent market failure of plazomicin illustrates that novel antibacterials with marketing approval for small patient populations affected by severe, MDR infections are not commercially viable within the current user-pays funding model.^20^ Inability to access life-saving antimicrobials is frequently reported in low- and middle-income countries;^21–23^ however, delays in access to new antimicrobials has also been highlighted as an issue in high-income countries such as Canada, Japan and many European countries.^10^

Globally, the death rate from lack of access to antibacterials is greater than the estimated death rate from AMR.^24^ Shortages of first-line antibacterials have been an ongoing problem in Australia, as well as internationally, and can lead to the inappropriate use of broader-spectrum drugs, more toxic or less effective antibacterials, which may have adverse impact on the patients and also on local resistance rates. In addition, if the shortage is extended, prescribers may change their prescribing habits due to the prolonged period of inability to comply with recommended guidelines. This is also true for unregistered products where the time delay to access the antibacterial may be considerable, requiring less effective alternatives to be used in the meantime. Cost has also been shown to be a barrier to using optimal treatment.

It is noteworthy that two of the most frequently accessed unregistered antimicrobials were for the salvage treatment of *H. pylori* infections. *H. pylori* resistance is increasing globally^25,26^ and a recent Australian study found that of 796 isolates isolated from 707 patients, 63% were MDR.^27^ Chinese manufacturer TenNor Therapeutics currently has a novel antibiotic targeting *H. pylori* infections in Phase III trials.^28^ If rifasutenizol reaches the marketplace it will be the first new antibacterial developed specifically for *H. pylori* infections in more than 30 years, and has only been possible with more than US$42 million in private investment, including money from the AMR Action Fund.^29^ The AMR Action Fund is a global public–private partnership, established in 2020, as an investment fund to stimulate the development of new antimicrobials.^30^

Just over 10% of SAS applications between 2018 and 2023 were via the Category B pathway. Unlike Category A or C, access to antibacterials via this pathway requires an approval letter prior to commencing treatment. Ninety-seven percent of Category B applications were approved within 20 days; however, approximately 1% took over 2 months for approval to access the antibacterial. Prescribers are required to justify the request, including a statement as to why currently available options are not appropriate. None of the Category B applications for antibacterials were declined during this period; however, the protocol for decision-making on these applications is not publicly available.

While timely access to optimal antibacterial treatment is essential, it is also paramount that new antibacterials are not overused or used inappropriately. Following the registration of oral fosfomycin in Australia there were concerns raised by infectious disease physicians due to the increase in use that was not in concordance with clinical guidelines. Fosfomycin is a last-line, broad-spectrum antibacterial reserved for urinary tract infections caused by pathogens resistant to all first-line antibacterials; however, it was initially marketed as a possible treatment option for all UTIs. This example illustrates the economic dichotomy of antibacterials—increased sales are essential for financial viability of the product; however, increased sales may also drive AMR. Stewardship measures aimed at reducing usage, to reduce the risk of AMR, also reduce the economic returns for companies. For this reason, alternative funding mechanisms, where reimbursement is de-linked from sales volume are being explored internationally in order to secure sustainable markets for antibacterials.^4,31,32^ Most notably in the UK, a fully de-linked subscription-style reimbursement model has been successfully piloted in England and is now being expanded throughout the UK.^33^ The UK Government has committed to evaluating and paying for selected antimicrobial products via a different method to other medicines, creating sufficient commercial incentives for manufacturers while supporting good stewardship.^33^ Although Australia is a relatively small market globally, as a high-income country there is opportunity to explore collaborations with other countries to help improve the supply chain insecurity, for both new and older antibacterials. A number of antimicrobials have been withdrawn from the Australian market, for example tinidazole and imipenem/cilastatin, due to insufficient sales due to relatively low volumes and short treatment courses. These examples illustrate opportunities where government policy change could support manufacturers to retain the registration within Australia and therefore reduce the risk of inaccessibility for patients. Waiving regulatory fees for low-volume or new antibacterials, implementing de-linked reimbursement and providing incentives to strengthen manufacturing capacity in Australia are possible avenues to ensure equitable access and improve the security of the supply chain.

In a positive step forward to improving equity of access to new antimicrobials for Australians, in 2024 the Australian Government released the final report of the Health Technology Assessment (HTA) Policy and Methods Review, which contained a number of recommendations that aim to incentivize the development and market entry of health technologies that address AMR.^34^ The recommendations included the exemption of regulatory fees for antimicrobial agents that target organisms on the WHO bacterial or fungal priority pathogen lists.^35,36^ The report also recommended that the Australian Government should develop a flexible reimbursement policy for antimicrobial products, and in the short term, pilot and assess a subscription fund for novel antimicrobials. The intention of the government is to implement HTA reform based on the recommendations of the report.

There are some limitations to the data provided here on the use of unregistered antimicrobials in Australia; no single dataset completely represents the use of all unregistered antimicrobials as each has different scope and limitations. SAS data from the Department of Health and Aged Care are a count of the individual requests to access an unregistered antimicrobial but do not accurately reflect the specific quantity used or duration of treatment. In addition, a single patient may require more than one course of treatment, and possibly multiple SAS applications, therefore the sum of the applications may not necessarily equal the number of individual patients accessing a drug. The NAUSP dataset reflects the amount (total grams) of antimicrobials dispensed or distributed for inpatient use, which is converted into a standardized metric, DDD per 1000 occupied bed days. This metric is a surrogate measure for actual consumption in Australian hospitals and excludes paediatric use due to DDD being an adult metric. Outpatient use is excluded from the NAUSP dataset. In addition, it is a voluntary surveillance programme and does not include all Australian hospitals, although all tertiary hospitals do contribute data to NAUSP. Data on clinical indications for which unregistered antimicrobials were used for were not available for this study.

### Conclusions

In Australia, the legislated access pathway for health providers to access unregistered antimicrobials for patients is utilized extensively, including for the treatment of life-threatening infections. These data illustrate the need that is currently not met by registered antimicrobials. To protect the entire healthcare system, the Australian Government allocation of funding resources to address AMR needs to also consider ensuring the availability of antibacterial treatments for people infected with resistant organisms. Whilst the Australian market alone is insufficient to encourage the development of new antimicrobials, as a high-income country, Australia can play an important role in contributing to a global response to the AMR crisis by addressing regulatory and economic barriers to the registration of antibacterials needed for otherwise untreatable infections.

## Supplementary Material

dlae216_Supplementary_Data

## Supplementary data

Figure S1 and Tables S1 and S2 are available as Supplementary data at *JAC-AMR* Online.
