# Supplementary material for: Do policies that allow access to unregistered antimicrobials address the unmet need? Australia as a case study of a high-income country with universal healthcare
Source: JAC Antimicrob Resist. 2025 Feb 27;7(1):dlae216. doi: 10.1093/jacamr/dlae216 (PMC11878572; doi:10.1093/jacamr/dlae216)
Supplement: dlae216_Supplementary_Data [file dlae216_supplementary_data.docx]

**Supplementary data**

**Table S1: Number of category A^#^ applications for antimicrobials via the Special Access Scheme, 2018-2023**

^#^Category A = urgent access for a critically ill patient; *novel = marketed internationally between 2013-2023; old = marketed prior to 2013.

**Figure S1: SAS applications for new antibacterials, by Australian state/territory, 2018-2023**

| **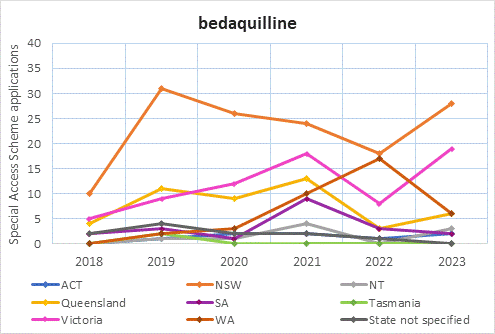** | **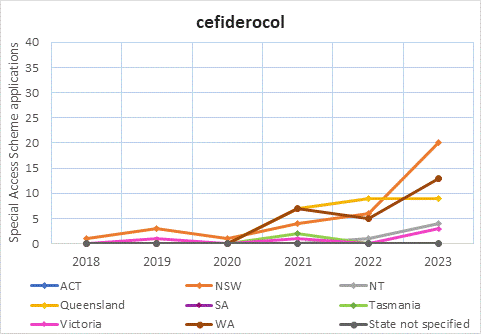** |
| --- | --- |
| **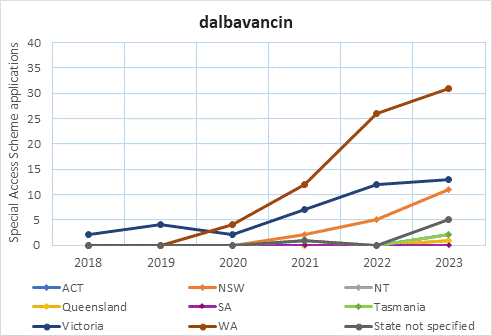** | **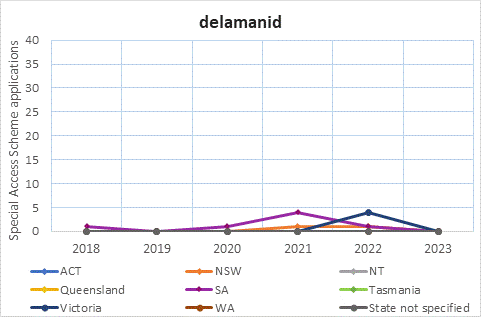** |
| **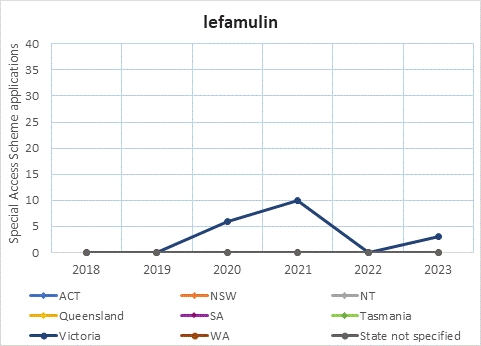** | **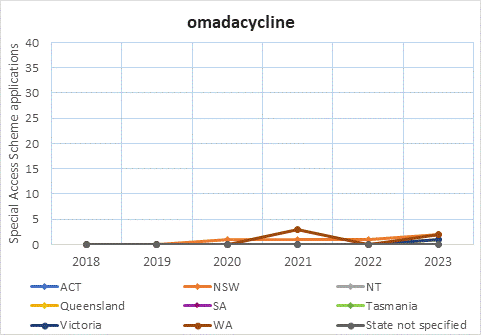** |
| **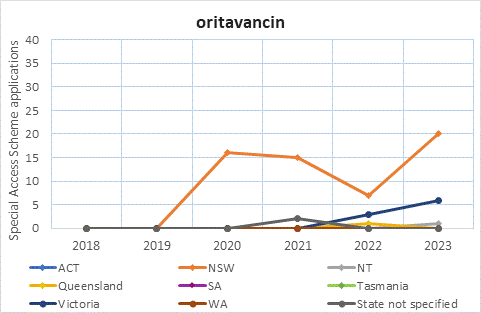** | **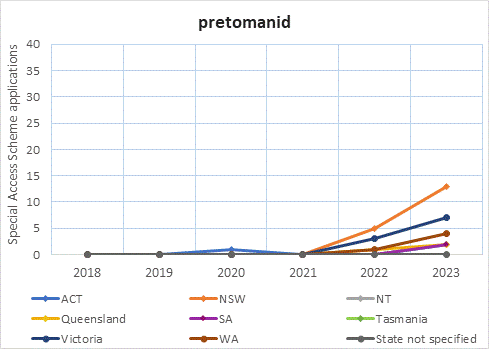** |

ACT = Australian Capital Territory; NSW = New South Wales; NT = Northern Territory; SA = South Australia; SAS = Special Access Scheme; WA = Western Australia

**Table S2: Reported use of new unregistered antibacterials in 2023: Comparison of the NAUSP and TGA (SAS) datasets**

| **Antibacterial** | **Dosage form** | **Use in Australia in 2023** | | | | | | | |  |  |
| --- | --- | --- | --- | --- | --- | --- | --- | --- | --- | --- | --- |
|  |  | **ACT** | **NSW** | **NT** | **Qld** | **SA** | **Tas** | **Vic** | **WA** |  |  |
| Aztreonam - avibactam | IV |  |  |  |  |  |  |  |  |  |  |
| Bedaquiline | oral |  |  |  |  |  |  |  |  |  |  |
| Cefiderocol | IV |  |  |  |  |  |  |  |  |  | Key: |
| Ceftobiprole | IV |  |  |  |  |  |  |  |  |  | No usage in SAS (TGA) or NAUSP data |
| Contezolid | oral |  |  |  |  |  |  |  |  |  |  |
| Dalbavancin | IV |  |  |  |  |  |  |  |  |  | Use reported to NAUSP and SAS |
| Delafloxacin | oral |  |  |  |  |  |  |  |  |  |  |
| Delafloxacin | IV |  |  |  |  |  |  |  |  |  | Use reported to SAS but not NAUSP |
| Delamanid | oral |  |  |  |  |  |  |  |  |  |  |
| Eravacycline | IV |  |  |  |  |  |  |  |  |  | Use reported to NAUSP but not SAS |
| Imipenem - cilastatin - relebactam | IV |  |  |  |  |  |  |  |  |  |  |
| Lascufloxacin | oral |  |  |  |  |  |  |  |  |  |  |
| Lefamulin | oral |  |  |  |  |  |  |  |  |  |  |
| Lefamulin | IV |  |  |  |  |  |  |  |  |  |  |
| Meropenem - vaborbactam | IV |  |  |  |  |  |  |  |  |  |  |
| Omadacycline | oral |  |  |  |  |  |  |  |  |  |  |
| Omadacycline | IV |  |  |  |  |  |  |  |  |  |  |
| Oritavancin | IV |  |  |  |  |  |  |  |  |  |  |
| Plazomicin | IV |  |  |  |  |  |  |  |  |  |  |
| Pretomanid | oral |  |  |  |  |  |  |  |  |  |  |
| Sarecycline | oral |  |  |  |  |  |  |  |  |  |  |
| Sulbactam - durlobactam | IV |  |  |  |  |  |  |  |  |  |  |
| Tedizolid phosphate | oral |  |  |  |  |  |  |  |  |  |  |
| Tedizolid phosphate | IV |  |  |  |  |  |  |  |  |  |  |
| Telavancin | IV |  |  |  |  |  |  |  |  |  |  |

ACT = Australian Capital Territory; NAUSP = National Antimicrobial Utilisation Surveillance Program; NSW = New South Wales; NT = Northern Territory;

SA = South Australia; SAS = Special Access Scheme; Tas = Tasmania; TGA = Therapeutic Goods Administration; Vic = Victoria; WA = Western Australia
